# The relational effects of perceived organizational support, fear of COVID-19, and work-related stress on the safety performance of healthcare workers

**DOI:** 10.3389/fpsyg.2022.963683

**Published:** 2022-10-10

**Authors:** Foluso Philip Adekanmbi, Wilfred Isioma Ukpere, Lovlyn Ekeowa Kelvin-Iloafu

**Affiliations:** ^1^Department of Industrial Psychology and People Management, College of Business and Economics, University of Johannesburg, Johannesburg, South Africa; ^2^Department of Management, Faculty of Business Administration, University of Nigeria, Enugu Campus, Nsukka, Nigeria

**Keywords:** sustenance, healthcare, impact, safety performance, COVID-19, Nigeria, stress

## Abstract

This paper assesses the relational effects of perceived organizational support (POS), fear of COVID-19 (FOC-19), and work-related stress (WRS) on the safety performance of healthcare staff. The sample for this research was extracted from the University College Hospital (UCH) in the Oyo State of Nigeria. The participants were midwives, doctors, auxiliary services staff, and nurses who functioned in a COVID-19 hospital ward, fever or respiratory ICU, Auxiliary services, or outpatient clinics. This investigation espoused a clinical cross-sectional survey involving self-reported surveys. Of the 150 questionnaires distributed, 147 were fit for scrutiny and analyzed with Statistical Packages for the Social Sciences (SPSS version 28). This paper established a relationship between POS and safety performance (SP). Besides, it showed a significant positive correlation between FOC-19 and SP. It further noted that work-related stress negatively relates to safety performance. Moreover, this study showed the significant joint strong influence of POC, FOC-19, and WRS on the safety performance of healthcare workers. Hence, healthcare institutions are encouraged to create adequate support for healthcare workers, particularly during a global health crisis. Government and healthcare institutions should also develop an awareness program on the danger and consequences of getting infected by the virus or infecting other significant others. This will increase the fear of COVID-19 and, consequently, health workers’ safety performance. Besides, it is recommended that the management of healthcare institutions provides a proper work structure and schedule to help reduce workloads, consequently reducing WRS, as lowering it improves healthcare workers’ safety performance.

## Introduction

Earlier studies noted safety performance in terms of rules and activities in enriching safety measures in an organization (Xia et al., 2018). Mostly, adherence or less devotion to these rules and actions are self-reported (Andersen et al., 2018). Researchers have further noted safety performance as the level of safety in an organization. They posited that safety performance is the actions that workers exhibit in their places of work to foster the safety and health of clients, employees, the public, and the environment (Fernández-Muñiz et al., 2017; Jahangiri et al., 2017; Gunduz and Laitinen, 2018). It could also mean the propensity of incidents that result in injuries, fatalities, and destruction of property (Erdogan et al., 2018). Safety performance involves behaviors like safety participation and compliance (Sampson et al., 2014; Zhu et al., 2020). Safety compliance refers to behaviors fundamental to the required safety behaviors to be completed by a worker to ensure a protected workplace (Griffin et al., 2000), for example, personal protective equipment. Safety participation can be described as voluntary behaviors for ensuring safety behaviors (Griffin et al., 2000), such as engaging in whistleblowing and attending safety meetings. Safety performance could be measured proactively or reactively. The proactive measure assesses the organization’s progress in instructing safety practices to improve performance. The reactive measure is the number of accident-free days or safe work records (Fogarty and Shaw, 2010; Atak and Kingma, 2011). The current investigation operationalizes safety performance through proactive measures.

POS is the “*employees’ beliefs concerning the extent to which the organization values their contribution and cares about their well-being*” (Eisenberger et al., 1986, p. 501). Thus, the workers’ perceptions that their wellbeing is valued include two denotations. The first is that the organization knows and appreciates its actions, whereas the second denotes the workers’ awareness that their wellbeing is vital to the organization (Ahmed and Nawaz, 2015). For healthcare workers to perform their most acceptable duties, they require great engagement, attainable by affording essential organizational support and an appropriate work atmosphere (Gupta et al., 2016). Notably, the motivation and retention of healthcare workers have developed into the foremost concern for policymakers and hospitals. Consequently, to improve the wellbeing and performance of healthcare workers, managers and healthcare organizations can apply several human resources management (HRM) exercises related to organizational support (Munir et al., 2016).

The Coronavirus Disease-2019 (COVID-19) was identified first in November 2019 in Wuhan, China (World Health Organization (WHO), 2020). In a nation such as Nigeria, the COVID-19 virus brings distinctive challenges to its health sector (Ogunwale et al., 2020). However, there are limited findings on the physical, psychological, and wellbeing impacts of plagues of transmittable diseases on healthcare workers (HCWs), mainly in terms of excessive workload, strain, and burnout linked to the danger of contamination (Xiao et al., 2020).

During a pandemic, healthcare workers’ essential role is massive, making them more vulnerable to strain and increasing their fear of getting infected (Panagioti et al., 2018). Moreover, the strain on HCWs during a pandemic worsens their mental performance and threatens their safety. The healthcare staff’s main concern is the danger of getting infected or spreading the virus to their relatives (Temsah et al., 2020).

Consequently, knowing the impact of organizational support, work stress, and the FOC-19 pandemic on HCWs’ wellbeing performance is fundamental to instituting mediations and policies to encourage the safety of healthcare workers. Researches on the relational effects of organizational support, work-related stress, and the FOC-19 on health experts’ safety performance in Nigeria are significantly few. This paper is important as it will add to the literature on safety performance, organizational and employee development, work stress, and psychology by providing evidence on how organizations can attain more safety performance levels and decrease the challenges and consequences of the COVID-19 pandemic.

Having indicated the proposed contribution of this investigation to the healthcare sector concerning the COVID-19 pandemic, this research aims to increase the literature on improving safety performance within this sector. This will be achieved by examining the correlational impacts of POS, FOC-19, work-related stress, and safety performance to infer a practical model to inspire and grow SP in the COVID-19 pandemic.

## Literature review

This paper’s literature review looks into perceived organizational support, the FOC-19, WRS, and safety performance.

### POS and safety performance

This paper’s investigation is based on the theory that healthcare workers’ safety behavior is tied to the support they receive from the health institution. This postulation originated from the organizational support theory (Eisenberger et al., 1986, 1997), which postulates that employees develop universal beliefs regarding the degree to which the organization values their contribution and cares about their wellbeing. Employees perceive their organization as supportive when they are rewarded beyond their contractual agreements and have their wellbeing cared for (Boateng, 2014). Consequently, it is pertinent to assess if this theory explains the safety participation and compliance of employees such as healthcare workers. Qi et al. (2019) noted that POS directly impacts safety performance. This view was supported by Zhihong (2012) that there is a correlation between POS and safety performance in air control organizations. Consistent with Mearns and Reader (2008), organizations expressing care for their workers will experience an unanticipated advantage, such as improved safety participation and compliance. Also, Ayim Gyekye and Salminen (2007) noted that workers are logical beings who feel how valuable their organization is to them. Hence, they tend to return the support and kindness received from their organization by conforming to safety ethics and engaging in other safety behaviors to improve the protection management system. Furthermore, Qi et al. (2019) concluded that organizational support correlates with safety performance. Also, Ahmad et al.’s (2017) study implied a significant positive impact of POS on employees’ safety performance. Consequently, the following is postulated:

*H1*: Perceived organizational support significantly correlates with the safety performance of Nigeria’s healthcare workers during the COVID-19 pandemic.

### FOC-19 and safety performance

The protection motivation theory (PMT) postulated that fear and perceived vulnerability explain human health behavior (Rogers, 1983), such as safety behaviors. The fear of COVID-19 is an example of what motivates employees to protect themselves. Hence, explaining the impact of healthcare workers’ fear of COVID-19 on safety performance hinges on the protection motivation theory. Toker et al. (2015) noted that the fear of getting infected by diseases within healthcare institutions influenced the work behaviors of healthcare workers, especially their hygiene and protection practices. Sarwar et al. (2022) indicated that the FOC-19 increases healthcare employees’ perception of increased danger to their wellbeing, increasing their safety behaviors. Furthermore, Follmer et al. (2020) suggested that because of the adverse effect the stigma of getting infected with a disease or virus has on the wellbeing of healthcare workers, they increase their safety practices. Taylor et al. (2020) also corroborate that the COVID-19 pandemic leads to stigmatization and discrimination; hence, the fears about its infection and contagion increase the safety performance of front liners within healthcare institutions. Reinforced by the studies on the fear of COVID-19 and safety performance indicated above, the current research hypothesized that:

*H2*: The FOC-19 significantly relates to the safety performance of Nigeria’s healthcare workers during the COVID-19 pandemic.

### WRS and safety performance

The Job Demand-Control theory of work stress, according to Karasek (1979), is the link between psychological demands and control. Omar et al. (2011) opined that job demand is the stresses, conflicts, work overloads, and uncertainties around duties. The basis of this theory is that employees are stressed in the face of high job demand, as they only have little control to go through vigorous situations of meeting demands. Hence, when employees go through an increased workload with little control, it affects their work behaviors. This has inspired a question of how a theory such as the job demand-control theory could impact employee safety behavior, especially the healthcare workers faced with a high workload in the face of COVID-19. Previous studies have indicated a correlation between work stress and SP (Turner et al., 2012; Li et al., 2013). Also, Bronkhorst (2015) established that high work stress negatively impacts HCWs’ safety performance. Jung et al. (2020) corroborated this fact when they indicated that work stress influenced the safety behavior of construction workers. Studies have demonstrated the association of work-related stress with an increased risk of work-related accidents (Vecchio et al., 2011; Stenfors et al., 2013; Bergh et al., 2014). Finding suggests that high workload and lack of organizational support are linked to a higher possibility of injury in a work-related disaster (Julià et al., 2013). Besides, research has indicated that work-related stress negatively correlates with safe working practices, increasing the chance of a workplace accident (Hilton and Whiteford, 2010; Nahrgang et al., 2011). Existing evidence shows that work-related stress is associated with several health-related behavioral risks (Nomura et al., 2010; Silva and Barreto, 2012; Tsai, 2012). Following the reviewed relational impact of WRS on SP in Nigeria’s healthcare sector, this paper has proposed the following:

*H3*: Work-related stress significantly correlates with the safety performance of Nigeria’s HCWs during the COVID-19 pandemic.

Consequently, as indicated by the stated literature, the following proposition is expressed:

*H4*: POS, FOC-19, and WRS collectively impact the safety performance of Nigeria’s HCWs during the COVID-19 pandemic.

## Materials and methods

This research espoused a cross-sectional scientific investigation involving surveys, including 150 HCWs from the University College Hospital in the Oyo State of Nigeria. A previous study indicated an excess workload within the University College Hospital in the Oyo State of Nigeria (Talabi, 2003). Hence, the choice of hospital. Oyo state was the first to rank high in the noticed COVID-19 incidents throughout the study phase in July 2020 (Oyeniran and Chia, 2020). These facts suggested the focus on Oyo State and University College Hospital. UCH was significantly attending to COVID-19 incidences during the present study. Questionnaires were floated among participants who were either doctors, nurses, auxiliary services, or midwives who worked in ICU, outpatient clinics, fever clinics, auxiliary services, or the COVID-19 hospital ward. This action was taken to test this study’s postulations and gather data on healthcare workers’ views on POS, FOC-19, work-related stress, and safety performance. All respondents were enlisted through a purposeful and simple random sampling method, and all the respondents volunteered to participate in this study, where respect for ethical matters was guaranteed. Participants’ names and personal information were not disclosed, and their participation did not endanger work or persons. One hundred and forty-seven (147) surveys were retrieved and concluded appropriately. Data recovered were cleaned and analyzed with Statistical Packages for the Social Sciences (SPSS vs. 28). To limit the variations in responses caused by the instrument, this research conducted validity and reliability analyses to realize the measure’s legitimacy and local dependability.

One of the major causes of common method variance is obtaining the measures of both predictor and criterion variables from the same source. However, despite the advantage of getting information from different sources, it is not feasible to use in all cases. For example, examining the relationships between two or more employee job attitudes (for instance, perceived organizational support, fear of COVID-19, work-related stress, and safety performance) cannot obtain measures of these constructs from alternative sources (Podsakoff et al., 2003). Another issue is to link the different sources together, which could compromise the anonymity of the respondents and reduce their willingness to participate or change the nature of their responses. In addition, it can also result in the loss of information when data on both the predictor and criterion variables are not obtained. Besides, considerable time, effort, or cost could have made the study impossible or delayed (Lindell and Whitney, 2001; Podsakoff et al., 2003). So, because the present study used one source in getting information, the common method variance was controlled for by having respondents complete the measurement of the predictor variable using different response formats (for instance, 5-point, 6-point, and 7-point Likert scales). Engaging respondents also controlled it at different locations (e.g., work departments).

This paper’s survey has segments:

*Section A: Participants’ demographics.*

This part covers the respondents’ demographics: age, gender, level of education, department, marital status, profession, and working experience.

*Section B: Perceived Organizational Support (POS) Scale.*

The 16-item POS measure developed by Eisenberger et al. (1986) was adopted in this study. It had a reliability coefficient of 0.95. The measure has a 7-point Likert-type response layout. However, this paper realized a reliability coefficient of 0.89. A sample item for this measure is *“The organization strongly considers my goals and values.”*

*Section C: FOC-19 Scale.*

In measuring the current investigation’s FOC-19 (FCV- 19S), this paper espoused a 7-item measure from Monterrosa-Castro et al. (2020). One of the sample items listed is *“I am afraid of losing life because of COVID-19.”* This instrument includes a 5-point Likert scale. This instrument’s original reliability figure was 0.82. However, the current research attained a 0.97 reliability coefficient.

*Section D: WRS Scale.*

The current study implemented the survey on WRS from a set of instruments investigating stress syndrome in several work events by Monterrosa-Castro et al. (2020). It comprises 12 items and 6-Likert-type response sets. An example of the measured item is *“I am usually unable to fall asleep.”* Nonetheless, no studies writing psychometric evaluations were documented. Yet, the present paper realized a reliability coefficient of 0.95.

*Section E: Safety Performance Scale.*

This paper adopted the safety performance measure from Heier et al. (2021). The instrument contains 16 items, with 4 items for safety motivation, safety compliance, safety knowledge, and safety participation sub-scales. “*I feel that it is worthwhile to put in the effort to maintain or improve my personal safety*” is an example of items that measure safety motivation as part of safety performance. Also, “*I know how to perform my job safely*” is an example of an item that measures safety knowledge as part of safety performance. Nonetheless, the safety knowledge sub-scale showed an *α* = 0.80 coefficient, while a reliability coefficient of *α* = 0.85 was realized in this paper. The safety motivation sub-scale achieved an *α* = 0.72 coefficient, while a coefficient of *α* = 0.80 was attained in the recent research. An *α* = 0.84 coefficient was realized for the safety compliance sub-scale, while this paper discovered a coefficient of *α* = 0.82 coefficient. The safety participation sub-scale had an *α* = 0.76 coefficient, while this paper established an *α* = 0.81 coefficient. Each statement was responded to using a 5-point Likert-type answer scale.

However, this paper conducted pilot research to authenticate the measure’s efficiency and realize the scale’s local reliability. A pilot study was also undertaken to identify likely problems earlier.

## Results

The data acquired from the participants were analyzed, and the findings are presented in Supplementary Table S1.

A total of 147 of 150 HCWs concluded the survey. Of those, 58 (39.5%) were male and 89 (60.5%) were female. Twenty-nine (29–19.7%) of these HCWs were between the age of 20–29, 45 staff (30.6%) and between 30 and 39, 43 (29.3%) between 40 and 49 years of age, and the other 30 (20.4%) were 50 years and above. Furthermore, 43 (29.3%) of the respondents were single, while the other 104 (70.7%) were married. Also, 29 (19.7%) of the HCWs had a college degree, 43 (29.3%) had a Bachelor’s degree, and 75 (51.0%) of healthcare staff had a Master’s degree or above.

In addition, of the 147 healthcare employees that finished the survey, 58 (39.5%) were doctors, 59 (40.1%) were nurses and midwives, and 30 (20.4%) of the staff rendered auxiliary services. This paper also comprised necessary clinical units, with 30 (20.4%) answers from the fever clinic, after that, employees from COVID-19 hospital ward 43 (29.3%), 30 (20.4%) auxiliary services units, 29 (19.7%) from the ICU, then outpatient clinics 15 (10.2%). Besides, 14 (9.5%) HCWs had working experience of fewer than 2 years, 59 (40.2%) had between 2 and 5 years of working experience, and the other 74 (50.3%) HCWs had working experience of 5 years and above.

This research conducted correlation and linear regression analyses to test the stated propositions.

The resulting matrix in Supplementary Table S2 shows that POS has a significant positive connection with safety performance at (*r* = 0.994; *p* < 0.001). Hence, HCWs’ perception of organizational support in Nigeria increases their safety performance. Results also show that FOC-19 substantially relates to an employee’s safety performance (*r* = 0.901; *p* < 0.001). This implies that an upsurge in HCWs’ FOC-19 in Nigeria generates better safety performance. Moreover, the findings suggest that WRS is negatively associated with safety performance (*r* = −0.977; *p* < 0.001). This finding deduces that the HCWs’ WRS within Nigeria decreases their safety performance levels.

The results shown in Supplementary Table S3 indicated that POS, FOC-19, and WRS considerably and strongly jointly impact safety performance among HCWs in Nigeria (*R* = 0.994, *R*^2^ = 0.988, *F* = 3891.913, *p* < 0.001). This result infers that POS, FOC-19, and WRS accounted for 99% of the perceived variations in safety performance among HCWs in Nigeria. The remaining 1% was attributed to other variables not measured in this investigation.

The resulting matrix in Supplementary Table S4 shows that perceived organizational support positively impacts safety performance (*β* = 1.064; *p* < 0.001). Thus, perceived organizational support increases the safety performance of Nigeria’s healthcare workers during the COVID-19 pandemic. Findings also indicate that the fear of COVID-19 has a positive relational influence on safety performance (*β* = 0.315; *p* < 0.001). This implies that increased fear of COVID among Nigeria’s healthcare workers improved their safety performance during the COVID-19 pandemic. Moreover, the results suggest that work-related stress has a negative relational impact and negatively impacts safety performance (*β* = 0.585; *p* < 0.001). So, work-related stress reduces the safety performance of Nigeria’s healthcare workers during the COVID-19 pandemic era.

## Discussion

This research noted that POS has a significant and positive relational impact on the safety performance of Nigeria’s healthcare workers. This observation assumes that perceived organizational support, where an organization recognizes and appreciates employees’ actions and that their wellbeing is essential to the organization, increases the safety performance of Nigeria’s healthcare workers during the COVID-19 pandemic. This finding is coherent with previously observed evidence of a correlation between POS and SP (Zhihong, 2012). It also supports Qi et al. (2019) view that organizational support correlates with safety performance. The present finding further corroborates Ahmad et al.’s (2017) position that POS has a significant favorable influence on employees’ SP. Therefore, this paper has confirmed that POS significantly correlates with the safety performance of Nigeria’s HCWs. It also supports the position of the organizational support theory that healthcare workers’ safety behavior is tied to the support they receive from the health institution.

This research showed that FOC-19 has a significant and positive relational influence on the safety performance of Nigeria’s HCWs. The results added that the FOC-19 among HCWs in Nigeria significantly increased their safety performance. This evidence validates the position of (Toker et al., 2015) that the anxiety of getting infected by diseases within the healthcare institutions influenced the work behaviors of healthcare workers, especially their hygiene and protection practices. The current results further corroborate the submission of Sarwar et al. (2022). They indicated that the FOC-19 increases HCWs’ perception of increased danger to their wellbeing; hence, it influences an increase in their safety behaviors. Also, this paper supports the position of Follmer et al. (2020) that because of the adverse effect the stigma of getting infected with a disease or virus has on the wellbeing of healthcare workers, they increase their safety practices. They also corroborate Taylor et al. (2020) that the COVID-19 pandemic leads to stigmatization and discrimination; hence, the fears about its infection and contagion increase the safety performance of front liners within healthcare institutions. Thus, these findings have confirmed the hypothesis that the FOC-19 significantly relates to the SP of Nigeria’s healthcare workers. It has also corroborated the protection motivation theory (PMT) that fear and perceived vulnerability explains human health behavior, such as safety behaviors.

In addition, the results in this research implied that WRS has a significant and negative relational impact on the safety performance of Nigeria’s healthcare workers. This infers that HCWs’ WRS in Nigeria significantly decreases their safety performance. This result is coherent with a previous study that high work stress negatively affects healthcare workers’ safety performance (Bronkhorst, 2015). The current findings have confirmed the studies that have demonstrated the association of work-related stress with an increased risk of work-related accidents (Vecchio et al., 2011; Stenfors et al., 2013; Bergh et al., 2014). This paper also supports the previous studies that work-related stress is negatively correlated with safe working practices, increasing the chance of a workplace accident (Hilton and Whiteford, 2010; Nahrgang et al., 2011). Hence, this paper has confirmed the hypothesis that work-related stress significantly correlates with the safety performance of Nigeria’s healthcare workers. It also demonstrates the Job Demand-Control theory of work stress that there is a link between stresses, conflicts, work overloads, uncertainties around duties, and employee work behavior. Hence, the job demand-control theory impacts employee safety behavior, especially the healthcare workers faced with a high workload in the face of COVID-19.

**Figure 1:**
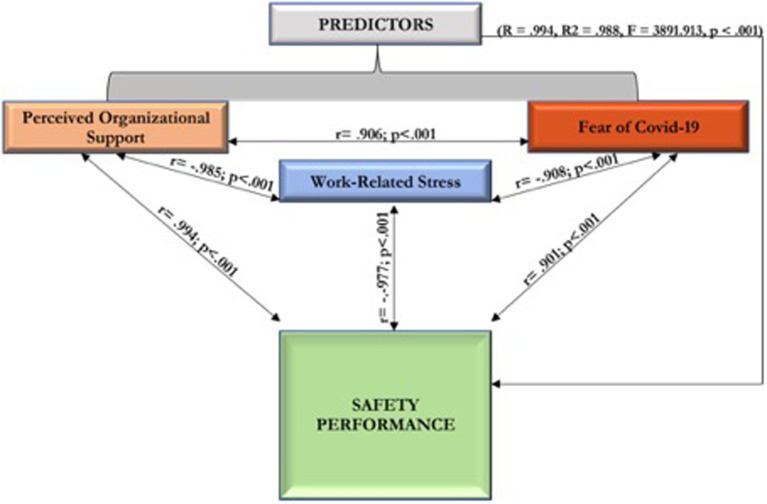
It presents the correlation matrix between the predictors and safety performance among HCWs in Nigeria.

This paper has further proven that POS, FOC-19, and WRS firmly and positively influenced the sustainability of safety performance among HCWs in Nigeria. Thus, these independent variables together induced a 99% variation in SP among the HCWs in Nigeria. The other 1% disparity in safety performance among healthcare workers in Nigeria is predicted by factors not measured in the current research. This result authenticates the hypothesis that POS, FOC-19, and WRS collectively impact the safety performance of Nigeria’s healthcare workers. Consequently, when Nigeria’s health sector carefully considers these variables in inspiring safety performance, much more results are achieved. The stated combined impacts of POS, FOC-19, and WRS on safety performance among HCWs in Nigeria have, therefore, been one of the significant and new contributions of this research. Consequently, Figure 1 presents the correlation matrix between the predictors and safety performance among HCWs in Nigeria.

**Figure 2:**
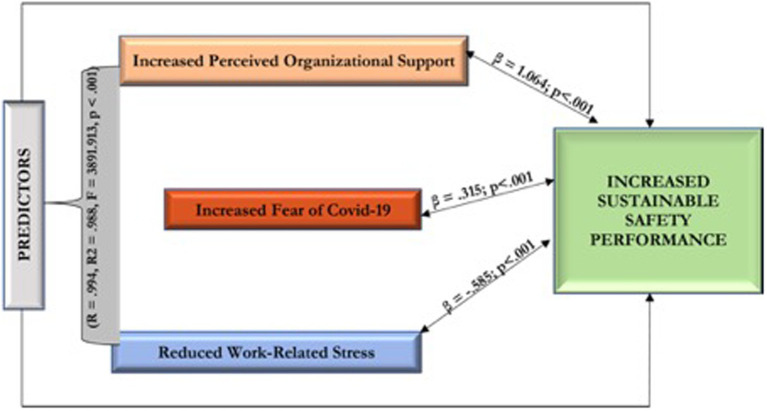
A practical model to inspire and sustain SP during the COVID-19 pandemic.

This research aimed to infer a practical model to inspire and sustain SP during the COVID-19 pandemic. Hence, the model is shown in Figure 2.

### Applied inferences

This paper’s results have several inferences for further study and appropriate systematic and developmental deliberate intervention necessary to achieve positive work outcomes, improved organizational support, the anxiety of susceptibility on the job, reduced work-related stress, and sustainable safety performance among HCWs. This paper’s results imply a significant improvement in safety performance within the healthcare sector by considering the combined effects of POS, FOC-19, and WRS. Likewise, these results will positively impact HCWs’ safety performance in Nigeria, specifically in the COVID-19 pandemic.

### Contribution

This paper adds to the literature on safety performance, organizational and employee development, work stress, and psychology. It also provides more new evidence (the relational effects of perceived organizational support, fear of COVID-19, and work-related stress) to how organizations can attain more safety performance levels and decrease the challenges and consequences of the COVID-19 pandemic.

### Limitation

This research was limited to a quantitative method. Hence, it could not provide a practical exploration to understand better factors predicting safety performance among HCWs in Nigeria in the face of COVID-19. Besides, this study was limited to University College Hospital (UCH) healthcare workers in the Oyo State of Nigeria. Hence, another limitation of the cross-sectional survey adopted by this research is generalizability (a limitation through a sampled population). Also, this study could not make a causal inference. Hence, because the exposure and outcome are simultaneously assessed, there is no evidence of a temporal relationship between exposure and outcome.

### Direction for future research

For future research into the impacts of predictors of safety performance among Healthcare Workers, researchers should adopt a mixed-method pragmatic study to explore the topic to have a broader knowledge of the influencers of safety performance during the COVID-19 pandemic. Besides, they could look into comparing States within a country like Nigeria. Future scholars should collect data from more than one study source to further reduce the potential influence of the common variance method (CVM).

## Conclusion and recommendation

This paper aimed to study how to promote and sustain HCWs’ safety performance in Nigeria, in the present COVID-19 pandemic, by examining POS, FOC-19, and WRS as predictors. Based on this paper’s results, perceived organizational support, FOC-19, and work-related stress have a convincing combined and independent impact on safety performance sustainability among HCWs in Nigeria. Thus, these stated independent variables predict safety performance among HCWs in Nigeria. Nonetheless, the following recommendations are helpful:

•This paper suggests the need for sufficient support for HCWs from their organizations, particularly throughout a global health crisis. This action will assist them in achieving and sustaining increased safety performance.
•Furthermore, the government and healthcare institutions should develop an awareness program on the danger and consequences of getting infected by the virus or infecting other significant others. This action will increase the FOC-19 and, consequently, HCWs’ safety performance.
•Also, the pandemic has caused a lot of tension and strain among healthcare workers. Hence, it is recommended that the management of healthcare institutions provide a proper work structure and schedule to help reduce workloads, consequently reducing WRS, as lowering it improves healthcare workers’ safety performance.

## Data availability statement

The raw data supporting the conclusions of this article will be made available by the authors, without undue reservation.

## Author contributions

FA: conceptualization, methodology, software, formal analysis, investigation, data curation, writing—original draft, and visualization. FA and WU: Validation and writing—review and editing. FA and LK-I: Resources. WU: supervision and funding acquisition. FA, WU, and LK-I: project administration. All authors contributed to the article and approved the submitted version.

## Conflict of interest

The authors declare that the research was conducted in the absence of any commercial or financial relationships that could be construed as a potential conflict of interest.

## Publisher’s note

All claims expressed in this article are solely those of the authors and do not necessarily represent those of their affiliated organizations, or those of the publisher, the editors and the reviewers. Any product that may be evaluated in this article, or claim that may be made by its manufacturer, is not guaranteed or endorsed by the publisher.
