# Supplementary material for: The relational effects of perceived organizational support, fear of COVID-19, and work-related stress on the safety performance of healthcare workers
Source: Front Psychol. 2022 Oct 10;13:963683. doi: 10.3389/fpsyg.2022.963683 (PMC9588951; doi:10.3389/fpsyg.2022.963683)
Supplement: Supplementary file 1 [file Data_Sheet_1.docx]

**Table 1**

**Descriptive Data of the HCWs’ demographics**

|  | | Freq. | % |  | | Freq. | % |
| --- | --- | --- | --- | --- | --- | --- | --- |
| Gender | Male | 58 | 39.5 | **Profession** | Doctor | 58 | 39.5 |
|  | Female | 89 | 60.5 |  | Nurse & Midwife | 59 | 40.1 |
|  | Total | 147 | 100 |  | Auxiliary Services | 30 | 20.4 |
| Age | 20-29 | 29 | 19.7 |  | Total | 147 | 100 |
|  | 30-39 | 45 | 30.6 | **Department** | Fever clinic | 30 | 20.4 |
|  | 40-49 | 43 | 29.3 |  | COVID-19 Hospital ward | 43 | 29.3 |
|  | 50 and Above | 30 | 20.4 |  | ICU | 29 | 19.7 |
|  | Total | 147 | 100 |  | Auxiliary services | 30 | 20.4 |
| Marital Status | Single | 43 | 29.3 |  | Outpatient clinics | 15 | 10.2 |
|  | Married | 104 | 70.7 |  | Total | 147 | 100 |
|  | Total | 147 | 100 | **Work Experience** | Less than 2 years | 14 | 9.5 |
| Highest Level of Education | College Degree | 29 | 19.7 |  | 2 – 5 years | 59 | 40.2 |
|  | Bachelor’s Degree | 43 | 29.3 |  | 5 years and above | 74 | 50.3 |
|  | Master’s Degree or Above | 75 | 51.0 |  | Total | 147 | 100 |
|  | Total | 147 | 100 |  |  |  |  |

**Source: Author’s Results**

**Table 2**

**The Zero-order correlations between the independent factors and the safety performance of HCWs in Nigeria**

| **Correlations** | | | | | | | |
| --- | --- | --- | --- | --- | --- | --- | --- |
|  | | **POS** | | **FO-19** | | **WRS** | **Safety Performance** |
| **Perceived Organizational Support** | Pearson Correlation | | 1 | | .906^**^ | -.985^**^ | .994^**^ |
|  | Sig. (2-tailed) | |  | | <.001 | <.001 | <.001 |
|  | N | | 147 | | 147 | 147 | 147 |
| **Fear of Covid-19** | Pearson Correlation | | .906^**^ | | 1 | -.908^**^ | .901^**^ |
|  | Sig. (2-tailed) | | <.001 | |  | <.001 | <.001 |
|  | N | | 147 | | 147 | 147 | 147 |
| **Work-Related Stress** | Pearson Correlation | | -.985^**^ | | -.908^**^ | 1 | -.977^**^ |
|  | Sig. (2-tailed) | | <.001 | | <.001 |  | <.001 |
|  | N | | 147 | | 147 | 147 | 147 |
| **Safety Performance** | Pearson Correlation | | .994^**^ | | .901^**^ | -.977^**^ | 1 |
|  | Sig. (2-tailed) | | <.001 | | <.001 | <.001 |  |
|  | N | | 147 | | 147 | 147 | 147 |
| ****. Correlation is significant at the 0.01 level (2-tailed).** | | | | | | | |

**Source: Author’s Findings**

**Table 3**

**A multiple regression showing the combined influence of POS, FOC-19, and WRS on SP**

| **Model** | **R** | **R Square** | **Adjusted R Square** |  |  |
| --- | --- | --- | --- | --- | --- |
|  |  |  |  | **F** | **Sig** |
| 1 | .994^a^ | .988 | .988 | 3891.913 | <.001^b^ |

**a. Dependent Variable: Safety Performance**

**b. Predictors: (Constant), Perceived Organizational Support, Fear of Covid-19, Work-Related Stress,**

**Table 4**

**A linear multiple regression showing the relational impacts of POS, FOC-19, and WRS on SP**

| Influencers | B | *β* | *t* | Sig | 95.0% Confidence Interval for B | | *R* | *R*^2^ | F | P |
| --- | --- | --- | --- | --- | --- | --- | --- | --- | --- | --- |
|  |  |  |  |  | Lower Bound | Upper Bound |  |  |  |  |
| (Constant) | -4.704 |  | -.986 | .007 | -14.131 | 4.724 | .994^a^ | .988 | 3891.913 | < 0.01 |
| Perceived Organizational Support | .995 | 1.064 | 19.665 | .001 | .895 | 1.095 |  |  |  |  |
| Fear of Covid-19 | .027 | .315 | .661 | .010 | .053 | .106 |  |  |  |  |
| Work-Related Stress | -1.088 | -.585 | -.550 | .023 | -.024 | -.201 |  |  |  |  |
